# Cladribine tablets as therapy for advanced relapsing-remitting multiple sclerosis: a 4-year follow-up real-world, multi-center, retrospective, cohort study

**DOI:** 10.3389/fneur.2025.1626317

**Published:** 2025-07-03

**Authors:** Aleksandra Pogoda-Wesołowska, Adam Stępień, Marcin Wnuk, Monika Marona, Elżbieta Tokarz-Kupczyk, Karolina Piasecka-Stryczyńska, Konrad Rejdak, Anna Jamroz-Wiśniewska, Monika Adamczyk-Sowa, Katarzyna Kubicka-Bączyk, Iwona Kurkowska-Jastrzębska, Katarzyna Kurowska, Przemysław Puz, Alina Kułakowska, Monika Chorąży, Waldemar Brola, Halina Bartosik-Psujek

**Affiliations:** ^1^Department of Neurology, Military Institute of Medicine, Warsaw, Poland; ^2^Department of Neurology, Jagiellonian University Medical College, University Hospital in Krakow, Kraków, Poland; ^3^Department of Neurology, Poznan University of Medical Sciences, Poznan, Poland; ^4^Department of Neurology, Medical University of Lublin, Lublin, Poland; ^5^Department of Neurology, Faculty of Medical Sciences in Zabrze, Medical University of Silesia in Katowice, Katowice, Poland; ^6^Department of Neurology, Institute of Psychiatry and Neurology, Warsaw, Poland; ^7^Department of Neurology, Upper Silesian Medical Centre of the Silesian Medical University in Katowice, Katowice, Poland; ^8^Department of Neurology, Medical University of Bialystok, Białystok, Poland; ^9^Department of Neurology, Jan Kochanowski University, Kielce, Poland; ^10^Department of Neurology, Institute of Medical Sciences, University of Rzeszow, Rzeszów, Poland

**Keywords:** cladribine, relapsing-remitting multiple sclerosis, safety, efficacy, real world evidence

## Abstract

**Introduction:**

Cladribine tablets (CladT) are a high-efficacy disease-modifying therapy recommended for the treatment of relapsing–remitting multiple sclerosis (RRMS) particularly in early disease. This study is aimed to evaluate the long-term efficacy of CladT in population of Polish RRMS patients, with more advanced disease.

**Methods:**

This retrospective observational study included patients with RRMS who started CladT treatment between December 2019 and November 2023. Collected data included prior treatments, annualized relapse rate (ARR), magnetic resonance imaging (MRI) activity, Expanded Disability Status Scale (EDSS) score, no evidence of disease activity (NEDA-3), lymphocyte counts, and safety outcomes were collected.

**Results:**

Of the 230 patients (8.3% treatment-naïve, mean disease duration 9.2 years), follow-up data were available up to year 1 for 222 patients, year 2 for 154 patients, year 3 for 87 patients and year 4 for 31 patients. The ARR decreased from 1.42 at baseline to 0.26, 0.22, and 0.36 in years 1, 2, and 3, respectively. The proportion of relapse-free patients increased from 13.9% at baseline to 76.8% in year 1, 82% in year 2 and 75.4% in year 3 with no relapses reported in year 4. The proportion of patients with active MRI lesions declined from 90.4% at baseline to 36.3% in year 1, 25.2% in year 2, 45.9% in year 3 and 8.3% in year 4. Stable or improved EDSS was observed in 85.9% of patients in year 1, 80.8% in year 2, 73.7% in year 3 and 88.9% in year 4. NEDA-3 status was achieved in 47.4% of patients in year 1, 51.0% in year 2, 40.4% in year 3 and 71.4% in year 4. Adverse events were reported in 16.7% of patients in years 1–2 and in 6.3% of patients in year 3.

**Discussion:**

The results indicate that CladT is effective and safe in Polish patients with RRMS, characterized by high disease activity, delayed treatment initiation, and multiple number of prior therapies.

## 1 Introduction

Multiple sclerosis (MS) is a chronic neurodegenerative disorder of the central nervous system that progressively leads to neurological deficits (1). It is characterized by recurrent episodes of inflammatory demyelination in the brain and spinal cord (2, 3).

Cladribine is a synthetic purine nucleoside analogue that induces transient lymphocyte apoptosis and depletion (4). Cladribine tablets (CladT) 10 mg (3.5 mg/kg cumulative dose over 2 years), approved in the European Union in 2017 and in the United States in 2019 for the treatment of adult patients with highly active relapsing forms of MS, have now gained marketing authorization in over 80 countries (5–8). As of the end of June 2024, approximately 101,132 patients have received CladT, with 251,900 patient-years of exposure since its approval (9).

CladT is a highly effective disease-modifying therapy (DMT) that acts as an immune reconstitution therapy. Unlike most other high-efficacy DMTs that act via continuous immunosuppression, CladT is administered orally in 2 short treatment courses over 2 years (8–10 days annually) (10–12). While offering an advantage of only a few treatment days per year (8 to 10), it offers a sustained therapeutic effect that may persist for up to 4 years, with no need for further treatment during that period (10, 12–14).

CladT is effective in relapsing–remitting MS (RRMS), reducing relapse rates, MRI activity, and disability progression (15–17). In patients with a first clinical demyelinating event, it was shown to reduce the risk of conversion to clinically definite MS and has a favorable safety profile (14, 18). Early initiation - especially in treatment-naïve patients or those with limited prior DMT exposure is associated with better outcomes (12, 13, 19, 20). Therefore, CladT is recommended for patients with active RRMS, including those with one relapse and MRI activity within the past year (21–23).

At the time of data collection, in Poland, under the national drug program, CladT could be offered to MS patients in whom other DMTs had been ineffective, as defined by at least 1 clinical relapse in the previous 12 months and at least 1 new gadolinium-enhancing (Gd+) lesion or at least 2 new T2 lesions. In treatment-naïve patients, CladT might be prescribed to those with at least 2 clinical relapses in the previous 12 months and an Expanded Disability Status Scale (EDSS) score between 0 and 4.5, or to those with at least 2 relapses requiring steroid treatment and at least 1 new Gd + lesion or at least 2 new T2 lesions (24). Consequently, all treatment-naïve patients in our cohort had highly active disease and many exhibited rapidly evolving severe MS.

The profile of MS patients treated with CladT in Poland differs markedly from populations commonly described in previous studies, which often focus on individuals in earlier stages of the disease. Consequently, there is a need for real world evidence (RWE) data to evaluate the clinical efficacy of CladT in patients with more advanced MS. Furthermore, the long-term effectiveness of CladT, particularly in previously treated patients, remains underexplored. This study was designed to address these gaps by assessing the long-term efficacy of CladT in a population of Polish RRMS patients with advanced disease and extensive prior treatment history.

## 2 Materials and methods

### 2.1 Study design

This retrospective observational study was conducted at 10 MS clinical centers in Poland and involved a cohort of all patients with RRMS who started treatment with CladT between December 2019 and November 2023.

One treatment course consisted of 2 cycles. All diagnoses were made according to the McDonald criteria (2017 update) (25). The study was approved by the Ethics Committee of the Polish Military Medical Chamber (approval number 235/22).

### 2.2 Data collection

The following data were collected: demographic characteristics; disease duration; number of previous MS therapies; the last DMT used before starting CladT treatment and the reason for switching; number of relapses in the past 12 months before CladT initiation and at 12, 24, 30, 36, 42, and 48 months after starting treatment; EDSS scores before CladT initiation and at 12, 24, 30, 36, 42, and 48 months; lymphocyte count before CladT initiation and at 2, 6, 12, 14, 18, 30, 36, 42, and 48 months; MRI assessments in the past 12 months before CladT initiation and at 12, 24, 36, and 48 months; adverse events (AEs); history of COVID-19 infection and SARS-CoV-2 vaccination; and discontinuation of CladT treatment or change to another DMT within the first 2 years of treatment and in the third and fourth years of treatment.

The differences in the number of patients evaluated for various parameters at the same time point stem from instances where certain parameters could not be assessed for specific patients due to data unavailability. As a result, the analyses for those parameters at that time point may include differing patient populations.

### 2.3 Definitions

Active MRI lesions were defined as Gd + lesions or as new or enlarging T2 lesions. No evidence of disease activity (NEDA-3) was defined as no relapses, no disability progression, and no active MRI lesions. The denominator for percentage calculations in a given year includes the total number of patients who achieved NEDA-3 and those who did not, provided that at least one of the three component parameters (number of relapses, MRI activity, or EDSS assessment) was available and indicated failure to meet the NEDA-3 criteria.

According to previous reports (26), changes in EDSS scores were classified as improvement or worsening as follows: for patients with a baseline EDSS score of 0, a change of at least 1.5 points; for patients with a baseline EDSS score of 0.5 to 4.5, a change of at least 1 point; and for patients with a baseline EDSS score of 5 or higher, a change of at least 0.5 points. EDSS changes that did not meet the criteria for improvement or worsening were classified as stable EDSS.

The degrees of lymphopenia were defined as follows: grade I (<1.0–0.8 × 10^9^/L); grade II (<0.8–0.5 × 10^9^/L); grade III (<0.5–0.2 × 10^9^/L); and grade IV (<0.2 × 10^9^/L) (27). The incidence of lymphopenia was assessed in patients whose lymphocyte counts were measured 2 months after the first treatment cycle or later, considering the lowest lymphocyte count recorded for each patient.

### 2.4 Statistical analysis

Descriptive data were presented as means and standard deviations (SDs) or medians and interquartile ranges (IQRs). The annualized relapse rate (ARR) with 95% confidence intervals (CIs) was calculated using a negative binomial regression model for the 12 months before CladT initiation and for the first, second, third-, and fourth years following treatment initiation. To compare ARR and EDSS results in years 1, 2, 3, and 4 of CladT treatment with the results before treatment initiation, the Wilcoxon test was used. A *p*-value of less than 0.05 was considered significant. Analyses were conducted using IBM SPSS Statistics (version 24.0.0.1) and the R (version 4.3.3) software.

## 3 Results

### 3.1 Patient characteristics

**Figure 1:**
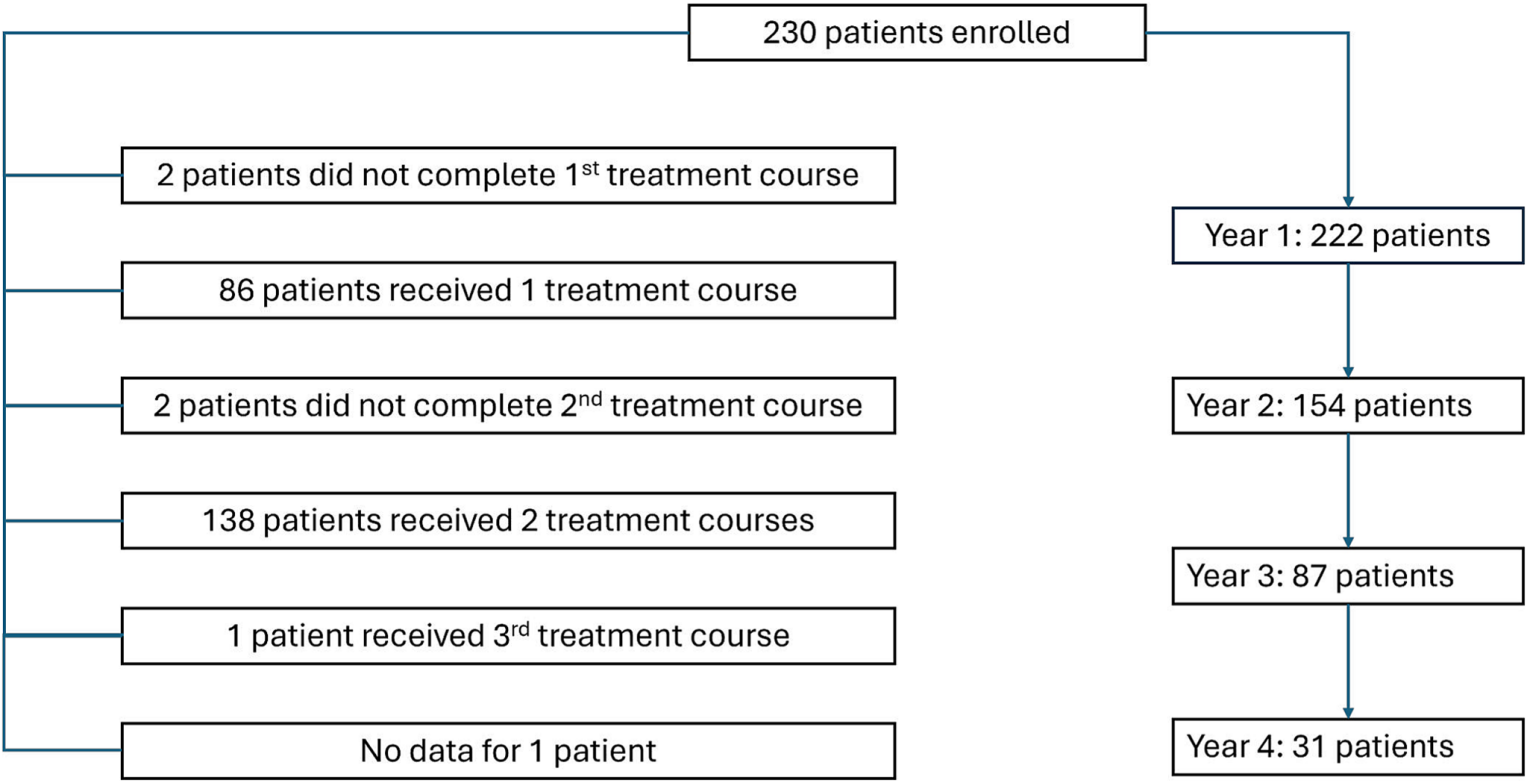
Flow diagram showing disposition of patients enrolled in the study.

Overall, 230 patients were included in the study, and follow-up data were available 1 for 222 patients at year, 2 for 154 patients at year 2, for 87 patients at year 3, and for 31 patients at year 4 (Figure 1) with median follow-up 24 months. Women constituted 77.8% of patients. The mean age of patients was 37.7 years (SD, 10.7 years). Until 2023, the mean disease duration was 9.2 years (SD, 5.9 years). The mean time between diagnosis and treatment initiation was 1.4 years (SD, 3.8 years), between treatment initiation and switching to CladT – 6.1 years (SD, 4.5 years), and between diagnosis and switching to CladT – 7.5 years (SD, 5.9 years). Only 8.3% of patients were treatment-naïve and most of them (11/19) had rapidly evolving severe disease. Before switching to CladT, most patients (69.2%) received 1 or 2 other DMTs, and the remaining 22.6% of patients received 3 or more DMTs. The most frequent DMTs were dimethyl fumarate (44.1%), fingolimod (14.7%), and teriflunomide (10.9%). In most cases (87.2%), the reason for switching to CladT was the inefficacy of previous therapy. Most patients (60.0%) received the full treatment of 4 cycles, and 1 patient received 6 cycles. The remaining patients were in earlier stages of treatment (Figure 1; Table 1).

**Table 1:** Baseline characteristics of patients.

Characteristic	Value
Sex (male/female); % (*n*); *N* = 230	77.8%
Age (years); mean (SD); *N* = 230	37.7 (10.7)
Disease duration (years); mean (SD); *N* = 229	9.2 (5.9)
Disease duration to initiating CladT (years); mean (SD); N = 229	7.5 (6)
EDSS; median (IQR); *N* = 226	2.5 (1.5, 3.5)
ARR; mean (SD); *N* = 230	1.42 (0.9)
Patients with active MRI lesions; % (*n*); *N* = 229	90.4 (207)
Lymphocyte count; median (IQR); *N* = 227	1.54 (1.25, 2.0)
Patients vaccinated against COVID-19; % (*n*); *N* = 230	67.0 (154)
Patients who underwent COVID-19 infection; % (*n*); *N* = 230	29.6 (68)
Number of MS therapies applied before initiating CladT; % (*n*); *N* = 230	
0	8.3 (19)
1	39.6 (91)
2	29.6 (68)
≥3	22.6 (52)
DMT used before switching to CladT; % (*n*); *N* = 211	
Dimethyl fumarate	44.1 (93)
Fingolimod	14.7 (31)
Teriflunomide	10.9 (23)
Glatiramer	9.0 (19)
Natalizumab	9.0 (19)
Interferon beta-1a	3.8 (8)
Interferon beta-1b	3.8 (8)
Ocrelizumab	2.4 (5)
Ozanimod	0.9 (2)
Alemtuzumab	0.9 (2)
Peginterferon beta-1a	0.5 (1)
Reason for switching to CladT; % (*n*); *N* = 211	
Inefficacy	87.2 (184)
AEs	5.7 (12)
John Cunningham virus	5.2 (11)
Planned pregnancy	0.9 (2)
Secondary progressive multiple sclerosis	0.5 (1)
Patient decision	0.5 (1)
Number of treatment cycles; % (*n*); *N* = 230	
1	0.9 (2)
2	37.4 (86)
3	0.9 (2)
4	60.0 (138)
6	0.4 (1)
No data	0.4 (1)

EDSS, Expanded Disability Status Scale; ARR, annual relapse rate; MRI, magnetic resonance imaging; CladT, cladribine tablets; COVID-19, coronavirus disease-2019; DMT – disease-modifying therapy; AE, adverse events.

### 3.2 Treatment efficacy

**Figure 2:**
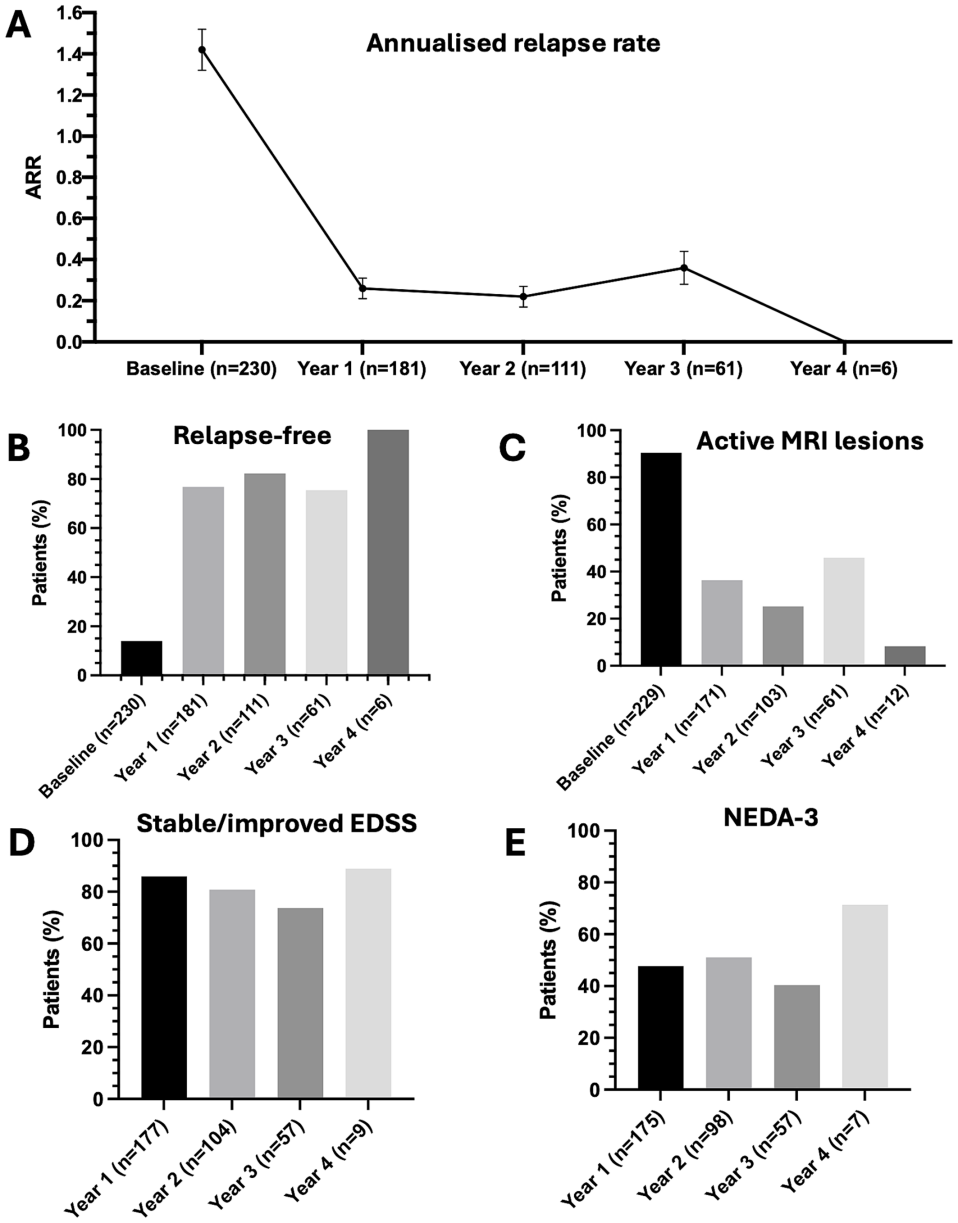
Efficacy outcomes after treatment with cladribine tablets. **(A)** Annualized relapse rate — point estimates are means and error bars are 95% confidence intervals. **(B)** Rate of relapse-free patients. **(C)** Rate of patients with active MRI lesions. **(D)** Rate of patients with stable/improved EDSS. **(E)** Percentage of patients with NEDA-3.

During CladT treatment, the ARR decreased from 1.42 (95% CI: 1.28–1.58) at baseline to 0.26 (95% CI: 0.2–0.35) in year 1 (*p* < 0.001), 0.22 (95% CI: 0.15–0.32) in year 2 (*p* < 0.001), and 0.36 (95% CI: 0.24–0.55) in year 3 (*p* < 0.001; Figure 2A). The proportion of relapse-free patients increased from 13.9% at baseline to 76.8% in year 1, 82% in year 2, and 75.4% in year 3 (Figure 2B). There were no relapses in year 4. The proportion of patients with active MRI lesions decreased from 90.4% at baseline to 36.3% in year 1 and 25.2% in year 2, increased slightly to 45.9% in year 3, and decreased to 8.3% in year 4 (Figure 2C). Compared with baseline, the median EDSS score did not change in year 1 (2.5 [IQR, 1.5–4.0], *p* = 0.73) and year 2 (3 [IQR, 1.5–4.0], *p* = 0.25), but changed significantly in year 3 (3.0 [IQR, 2.0–4.0], *p* < 0.01). Stable or improved EDSS scores were observed in 85.9% of patients in year 1, 80.8% of patients in year 2, 73.7% of patients in year 3, and 88.9% of patients in year 4 (Figure 2D). NEDA-3 was achieved in 47.4% of patients in year 1, 51.0% of patients in year 2, 40.4% of patients in year 3, and 71.4% of patients in year 4 (Figure 2E).

### 3.3 Safety

The median lymphocyte count decreased from 1.54 at baseline to 0.87 at month 2 and 0.71 in month 14. It subsequently started to increase and reached the baseline level by month 42 (Supplementary Figure 1).

Lymphocyte counts were within the reference range for 15.6% of patients, while 19.3% had grade 1 lymphopenia, 43.6% had grade 2 lymphopenia, 20.6% had grade 3 lymphopenia, and 0.9% had grade 4 lymphopenia. In years 1 and 2, lymphopenia occurred in 29.7% of patients. Grade 3 and 4 lymphopenia was reported only through month 18. The rates of lymphopenia during the study are presented in Supplementary Figure 2. Overall, AEs occurred in 37 patients (16.7%) in years 1 and 2 and in 4 patients (6.3%) in year 3. There were no AEs in year 4. Fatigue, herpes zoster infection, headache, and urinary tract infections were observed in 8.1% of patients each. Elevated liver enzymes, herpes simplex infection, and nausea were recorded in 5.4% of patients. There were also single cases of unspecified infections and COVID-19, eyeball pain, recurrent herpes simplex infection, drowsiness, skin rash, and dizziness.

Overall, 77.4% of patients completed or continued CladT treatment without switching to another therapy or starting another treatment during follow-up. The remaining 14.3% of patients discontinued treatment with CladT and/or switched to another DMT, while no data were available for 8.3% of patients (Table 2). Eleven patients (5%) discontinued treatment during years 1 and 2, 22% (17 of 79 patients) during year 3, and another 14% (5 of 36) during year 4. The reasons for discontinuation in years 1 and 2 were inefficacy (45.5%, 5 patients), patient decision (18.2%, 2 patients), and in 36.4% of cases (4 patients), the reason was unknown. In 30 patients, CladT was switched to another DMT, including ocrelizumab (50%), natalizumab (6.7%), fingolimod (3.3%), or mitoxantrone (3.3%). No data on subsequent treatment were available for 36.7% of cases. Among the 30 patients who switched from cladribine to another DMT, reasons for treatment discontinuation were documented only within the first 2 years of follow-up. Within this period, the reported reasons included lack of efficacy in 4 patients (13.3%), lymphopenia in 1 patient (3.3%), and a diagnosis of primary progressive multiple sclerosis in 1 patient (3.3%). In 5 cases (16.7%), no specific reason for discontinuation was recorded. In the remaining 19 patients (63.3%), cladribine treatment was not discontinued during the first 2 years, and therefore no data on the reasons for discontinuation were available for these individuals.

**Table 2:** Patient compliance with cladribine tablets treatment.

Switch or discontinuation; *N* = 230	% of patients (*n*)
No switch or discontinuation of treatment	77.4 (178)
Switch and/or discontinuation of treatment	14.3 (33)
No data	8.3 (19)
Discontinued treatment and/or switch	
Year 1–2; *N* = 211	5.2 (11)
Year 3; *N* = 79	21.5 (17)
Year 4; *N* = 36	13.9 (5)

## 4 Discussion

RWE data is important for understanding the effectiveness and safety of medications, as they better reflect clinical practice than clinical trials. This study presents long-term data on the efficacy and safety of CladT treatment in Polish patients with MS.

Due to reimbursement rules in Poland, the use of CladT in patients with early-stage disease was very limited. Although these criteria have evolved over time, they remained conservative at the time of data collection, restricting access to CladT treatment primarily to patients with highly active and advanced disease, for whom the potential treatment benefits are relatively limited. Consequently, CladT treatment was initiated much later in Poland compared to standard care. As a result, our study population was suboptimal for evaluating CladT treatment efficacy compared to most available studies. Recruited patients were characterized by relatively long disease duration, a prolonged interval before CladT initiation, a high baseline ARR, and a very low proportion of treatment-naïve individuals, with over 50% of patients having received 2 or more other therapies prior starting CladT. Highly effective treatments, including fingolimod, natalizumab, ocrelizumab, ozanimod, and alemtuzumab, were previously used in 28% of patients, with most switching to CladT due to inefficacy of previous therapy. Several authors reported better treatment outcomes in treatment-naïve patients and those switching from first-line therapies compared to those switching from second-line therapies (12, 13, 20, 28–30). Treatment-naïve patients with 2 relapses in the previous 12 months, as well as those with 1 relapse and 1 or 2 poor prognostic factors (such as age >40 years, male sex, smoking, relapse severity and high lesion load) were suggested as good candidates for CladT treatment in contrast to patients who had experienced more than 2 relapses within the last 12 months (21). Despite the suboptimal characteristics of our sample, CladT treatment proved to be highly effective: 2 years after treatment onset, 51.0% of patients achieved NEDA-3, 82% were free from relapses, 80.8% remained free from EDSS progression, and 74.8% had no disease activity on MRI. These results are consistent with other studies demonstrating the clinical efficacy of CladT (11, 13, 15, 19, 20, 23, 29, 31, 32).

In presented study, 77.4% of patients completed or continued CladT treatment without switching to another therapy during the follow-up period, which is in line with the CLASSIC-MS study (33). A total of 11 patients (5%) discontinued treatment in year 1 or 2, with 45.5% of these cases attributed to treatment inefficacy. Additionally, 22 patients switched to another treatment in years 3 and 4. The discontinuation and switching to other treatments can be explained by the different types of responses to CladT treatment demonstrated by our patients, as previously described. According to recommendations, patients showing greater disease activity after treatment initiation in year 1, as well as those with substantial activity in years 3 or 4, should be switched to an alternative therapy. However, patients showing moderate disease activity or stable disease in year 1, followed by moderate activity in year 2 or minor activity in years 3 or 4 should receive additional CladT courses rather than be switched to another therapy (34–36). Importantly, some of our patients started their treatment in 2019 and 2020, when clinical management during longer follow-up and responses to treatment had not yet been established. Current recommendations state that the full cumulative dose of CladT should be administered even if disease activity occurs between the first and second courses (36, 37). Furthermore, treatment efficacy should be assessed only after 2 full cladribine courses, typically no earlier than 14 months after treatment initiation (21). The reappearance of disease activity in year 3, as observed in our study, has also been reported by other authors (13, 38, 39). This response pattern may be characteristic of mid-term responders, according to the classification proposed by German experts (34, 35). In such cases, it is recommended to continue yearly follow-ups and either administer additional CladT courses (in cases of minor activity without safety concerns) or switch to another DMT (if significant disease activity is present) (34, 35).

To our knowledge, the presented analysis was the first RWE study in Poland and represented a continuation of our research, which was published in 2023 (40). As mentioned in the introduction, at the time of data collection in Poland, according to the drug program, CladT could be offered to MS patients who had failed other DMTs due to inefficacy or previously untreated patients with had highly active disease or even rapidly evolving severe form of the disease. As a result, the profile of MS patients treated with CladT in Poland differed significantly from the populations most described in the literature, which often focuses on patients in earlier stages of the disease. In our analysis patients were older, with a longer disease duration and mostly previously treated. Moreover, this study was characterized by longer follow-up period.

Similarly to our results, in the study conducted by Magalashvili et al., among 128 patients with highly active MS that received CladT treatment, clinical outcomes were assessed in 61 patients at year 3 and in 35 patients at year 4 (32). At treatment initiation, the mean age was 39.6 years, disease duration was 12.7 years, EDSS was 3.7, and the ARR was 1.6. In addition, in study by Liza et al. the patients included in the study were an older population with a higher disability rate (11). However, the study also included patients with secondary progressive MS (SPMS), and the follow-up time was shorter (2 years). Furthermore, results from the study by Santos et al. showed that mean disease duration at CladT initiation was 8.9 and most patients (86.1%) were not treatment-naïve (31). However, 88.5% patients were diagnosed with RRMS and 11.5% with SPMS.

In contrast, in the study of Pfeuffer et al. (12), patients had lower disease activity and a shorter disease duration. The follow-up period was also shorter than in our study. Moreover, among the patients included in the study by Zanetta et al. (13), half of the patients were treatment-naïve. Like our findings, the previously untreated group was characterized by higher disease activity, but the follow-up time was limited to only 25 months. In turn, in the study by Petracca et al. (20), untreated patients (29.3%) were significantly younger and had a shorter disease duration compared to those switching from other therapies (32.86 vs. 35.7 years, *p* = 0.02 and 1.54 vs. 8.74 years *p* < 0.0001, respectively) as in our study. However, treatment-naïve patients and previously treated patients did not differ for ARR, presence of active lesions, or EDSS at baseline. Additionally, observation period in that study was only 22 months.

There is a paucity of studies evaluating the long-term efficacy of CladT. Studies with follow-up of beyond 36 months have reported a decline in the proportion of NEDA-3 patients, relapse-free patients, and patients with stable EDSS score between years 2 and 5 (26, 38, 39, 41). Additionally, a recent meta-analysis of CladT efficacy showed consistently better outcomes in studies with less than 24 months of follow-up than in studies with longer follow-up (42). These findings suggest that some patients may need additional CladT courses beyond the standard 2 courses given in years 1 and 2, supporting the recommendations proposed by German experts (34, 35). Further long-term studies are needed to clarify this issue.

Overall, CladT was well tolerated and demonstrated a favorable safety profile, consistent with previous reports (13, 15, 23). As in earlier studies, more patients reported AEs in years 1 and 2 than in year 3 (12, 13). The most common AEs were headache, fatigue, infections, nausea, and elevated liver enzymes, in line with previous data (13, 19, 29, 31, 43, 44). No additional AEs, including serious AEs, were reported in our cohort. These findings support the favorable safety profile of CladT in a population characterized by older age, longer disease duration, and more extensive prior treatment than in most other studies.

Lymphopenia is an expected effect of CladT treatment due to its mechanism of action (44). The initial decrease in lymphocyte counts after each CladT course, followed by gradual recovery, represents a typical pattern of lymphocyte kinetics after CladT exposure (12, 13, 20, 45, 46). Consistent with other reports, peaks of lymphopenia appearing in months 2 and 14 were observed (12, 13). In our cohort, 20.6% of patients experienced grade 3 lymphopenia, which is in line with other studies (12, 15, 19, 47), although some authors reported lower rates (13, 20, 48). Grade 4 lymphopenia was rare and transient, with an observed rate of 0.9%, confirming earlier findings (12, 13, 15, 19, 20, 47, 48). Additionally, as observed in other studies, a higher rate of grade 3 and 4 lymphopenia in year 2 compared to year 1 was observed (45). It has been reported that lymphopenia was more common in previously treated patients than in treatment-naïve individuals (12, 13, 48), with a higher number of previous therapies being a predictor of grade 3 lymphopenia (13). Despite the high proportion of first- and second-line switchers in our population, our data suggested a safety profile comparable to that reported by other studies.

The main limitation of our study was the small number of patients over the long follow-up period. As the population of Polish patients treated with CladT increases over time, future studies should provide more insights into the long-term efficacy of CladT treatment in Polish patients with RRMS.

In conclusion, our results suggested that CladT was effective and safe in the Polish population of RRMS patients characterized by high disease activity, late treatment initiation, and multiple previous therapies.

## Data Availability

The raw data supporting the conclusions of this article will be made available by the authors, without undue reservation.
