# Supplementary material for: Cladribine tablets as therapy for advanced relapsing-remitting multiple sclerosis: a 4-year follow-up real-world, multi-center, retrospective, cohort study
Source: Front Neurol. 2025 Jul 3;16:1626317. doi: 10.3389/fneur.2025.1626317 (PMC12272060; doi:10.3389/fneur.2025.1626317)
Supplement: Supplementary file 1 [file Table_1.DOCX]

Supplementary Material


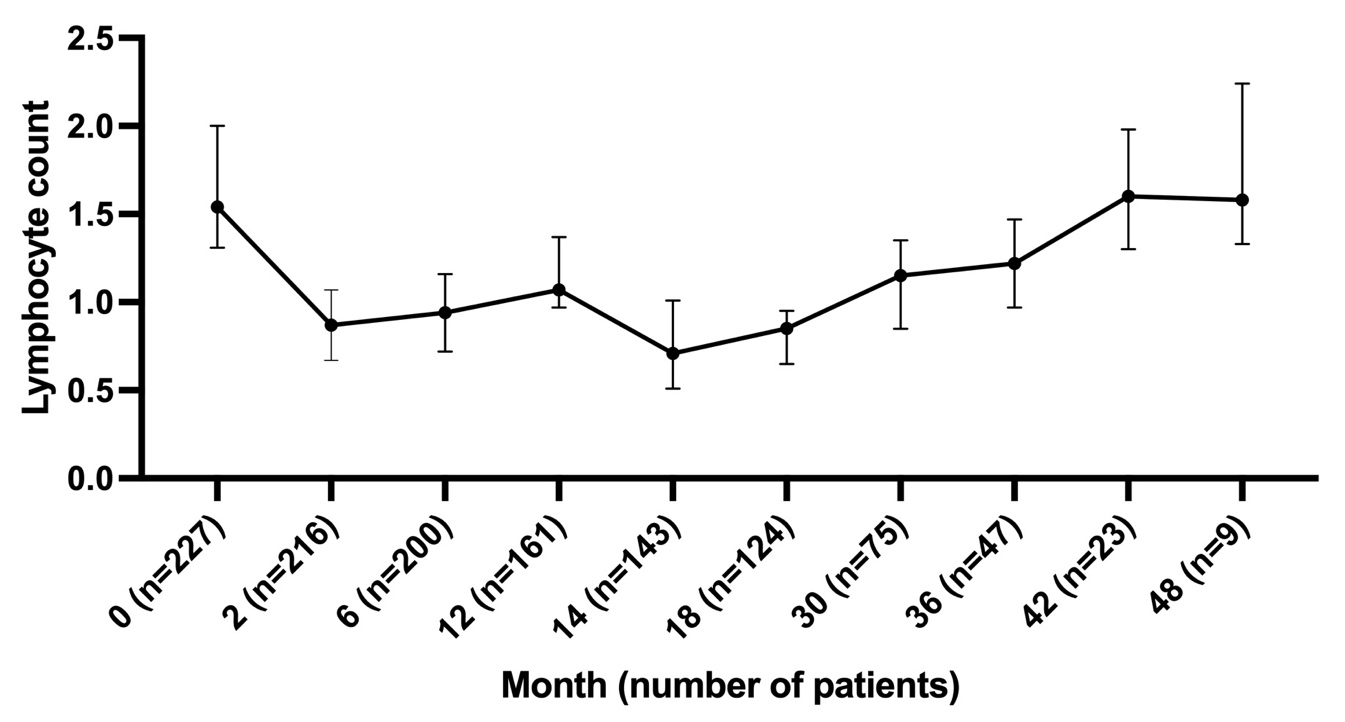


Supplementary Figure 1. Median lymphocyte count during the study

Supplementary Figure 2. Lymphopenia grades across time
